# Objective measurement of physical activity and sedentary behavior among South Asian adults: A systematic review

**DOI:** 10.1371/journal.pone.0236573

**Published:** 2020-08-05

**Authors:** Bushra Mahmood, Tricia S. Tang, Rowshanak Afshar, Maureen C. Ashe

**Affiliations:** 1 Department of Medicine, University of British Columbia, Vancouver, British Columbia, Canada; 2 Centre for Hip Health and Mobility, Vancouver, British Columbia, Canada; 3 Department of Family Practice, University of British Columbia, Vancouver, British Columbia, Canada; Washington State University, UNITED STATES

## Abstract

**Background:**

South Asians are one of the fastest growing ethnic groups in western countries with a high incidence of chronic diseases like metabolic syndrome and cardiovascular disease occurring at younger ages and lower body weight compared with white Europeans. Physically active lifestyle and reduced sedentary time are modifiable risk factors that can decrease burden of chronic diseases. Population-level surveys based on self-report show South Asians engage in low levels of physical activity. Because of known limitations with self-report data, we aimed to synthesize available evidence to generate a physical activity /sedentary time profile of South Asians from studies using accelerometry.

**Methods:**

We systematically searched Medline, EMBASE, CINAHL, PsycINFO, and SportDiscus. We included studies applying accelerometry to measure physical activity /sedentary time under free-living. Studies with an exclusive focus on drugs or including participants with health conditions/physical disability, and special populations (athletes/pregnant women) were excluded. Two authors independently adjudicated inclusion of citations at title/abstract and full text. We applied a standardized data abstraction form to extract relevant data. We evaluated methodological quality using Newcastle Ottawa Quality Assessment Scale. Due to variability and inconsistencies in measurement and reporting of physical activity /sedentary time, we only provide a narrative synthesis.

**Findings:**

We identified only 14 studies(n = 1,338). Despite using similar accelerometry assumptions, we noted variability in reported outcomes for physical activity and sedentary time. Sedentary time ranged from 482(98) to 587 min/day. Mean light physical activity ranged from 211.69(67. 38) to 574(227) min/day. Moderate to vigorous physical activity among South Asian women ranged from 17–41 min/day and among men, 32–43 min/day.

**Conclusion:**

South Asians exhibited higher levels of physical activity when compared to the Canadian population level survey but not when compared to the American population level survey. Overall, fewer studies, and small sample sizes led to considerable variability limiting any effective comparisons. Results highlight the importance of conducting methodologically robust studies based on random sampling to advance the field, and to capture true levels of sedentary time and physical activity in the South Asian population.

## 1. Introduction

South Asians, people belonging to or having ancestors from India, Pakistan, Bangladesh, Nepal, Bhutan, Sri Lanka or Maldives, are identified as a high-risk group for developing early onset of metabolic syndrome—a condition characterized by a cluster of cardiovascular disease risk factors including central adiposity, hypertension, type 2 diabetes mellitus (T2D) and dyslipidemia [1–3]. Prevalence of Type 2 diabetes (T2D), an underlying risk factor for coronary heart disease, is > 10% in many parts of the South Asian region [4]. In UK, Canada and the United States, T2D is 3–5 times higher in South Asian immigrants compared with other ethnic groups [1,2,5–7]. Multiple factors account for this increased metabolic risk among South Asians. A complex interaction between unhealthy diet and physical inactivity coupled with genetic predisposition places South Asians at a higher risk for cardiovascular disease at lower body mass index (BMI) and a relatively younger age compared with other ethnic groups [8–11].

Regular physical activity, defined as any bodily movement produced by the skeletal muscles that expends energy [12] is protective against many non-communicable diseases including hypertension, coronary heart disease, stroke, diabetes, breast and colon cancer [13–15]. In contrast, prolonged sedentary time, defined as any activity while sitting or in a reclined position that equates to less than or equal to 1.5 Metabolic Equivalents of Task (METS) [16], has been associated with a higher risk of developing metabolic syndrome [17] Despite this evidence, physical activity levels among South Asians, when compared with general population, remain low as reflected in population-level studies conducted in western countries. According to data from the Health Survey for England, South Asians were 60% less likely than native white population to meet recommendations of 150 minutes of moderate to vigorous physical activity (MVPA) per week [18]. Data from Canadian Community Health Survey revealed only 34% of South Asians were moderately active, with the lowest prevalence of moderate activity (12%) observed in South Asian women [19]. Similarly, in the US, the prevalence of physical inactivity was close to 60% in South Asian immigrants [20]. Within the South Asian region itself, a recent systematic review showed a wide variation in the prevalence of physical inactivity with >75% of South Asian adults observed to be inactive during leisure time [21].

Type of measurement can significantly impact the observed levels of physical activity. Most of the knowledge regarding low prevalence of physical activity in South Asians is from self-reported questionnaires. These measures have known limitations such as imperfect recall and social desirability bias, as well as difficulty in assessing accurate frequency, duration and intensity of activities [22]. This often results in over or under-estimation of sedentary time and true physical activity energy expenditure. Accurate assessment is imperative to measure current and changing sedentary time and physical activity levels, and to evaluate the effectiveness of interventions designed to increase activity levels [22]. Accelerometers provide objective estimates of sedentary time, physical activity, and sleep, and their use has increased dramatically [23]. But gaps remain for accelerometry assessment of sedentary time and physical activity for the South Asian population. Therefore, our purpose for this review was to (i) identify studies that used activity monitors for an objective measurement of sedentary time and various intensity physical activities (light, moderate, vigorous) and, (ii) create sedentary time and physical activity profiles for South Asians based on the number, quality and representativeness of available data.

## 2. Methods

This was a systematic review of evidence for accelerometry measurement of sedentary time and physical activity among South Asian adults aged ≥18 years. We conducted and reported this review using the preferred reporting items for systematic review/meta-analysis protocols (PRISMA) [24]. A protocol does not exist for the review. Prior to starting the review, we registered it with the International Prospective Register of Systematic Reviews (PROSPERO).

### 2.1 Eligibility

Our selection criteria included all studies with a South Asian adult population (≥18 years) that applied an accelerometer for characterizing sedentary time and/or various intensity physical activities. We limited our search to peer-reviewed studies with no restrictions on language, year of publication or geographic location. We included all study designs that provided accelerometry data for ≥ 3 days. We excluded studies with an exclusive focus on drugs/surgical interventions in combination with physical activity interventions (unless baseline data were reported), and studies reporting energy expenditure. We also exclude studies with special populations such as athletes, pregnant women, or participants with serious medical/chronic conditions and/or physical disability.

### 2.2 Information sources

We searched the following databases to identify studies that met our eligibility criteria: Medline (Ovid), PubMed, EMBASE (Ovid), the Cumulative Index to Nursing and Allied Health Literature (CINAHL), Cochrane Library (Cochrane Database of Systematic Reviews, Cochrane Central Register of Controlled Trials (CENTRAL), PsycINFO (EBSCO) and SportDiscus.

### 2.3 Search strategy

We developed a comprehensive search strategy, guided by a university librarian with content expertise in PA. We used a combination of Medical Subject Headings (MeSH) and free text words to search the identified electronic databases. Search strategy was first developed for Medline (Ovid) and was then tailored for each database. We provide the search for Ovid Medline in Box 1.

## Box 1. Ovid Medline search

1. asia, western/ or exp bangladesh/ or exp bhutan/ or exp india/ or exp afghanistan/ or exp nepal/ or exp pakistan/ or exp sri lanka/
2. exp Asian Americans/
3. exp Indian Ocean Islands/
4. (India* or punjab* or Pakistan* or Sri Lanka* or Bangladesh* or Nepal* or Maldives* or Bhutan* or Afghanistan* or Afghani*).mp. [mp = title, abstract, original title, name of substance word, subject heading word, keyword heading word, protocol supplementary concept word, rare disease supplementary concept word, unique identifier, synonyms]
5. exp "Emigrants and Immigrants"/
6. 1 or 2 or 3 or 4 or 5
7. exp exercise/ or physical endurance/ or physical exertion/ or exp physical fitness/
8. exp Sports/
9. exp running/ or jogging/ or swimming/ or exp walking/ or exp motor activity/
10. ("physical activit*" or (physic* adj3 activit*) or sport* or exercis* or walk* or (active adj3 (transport* or commut*)) or cricket or wrestling or hockey or swim* or bicycling or cycling or tennis or volleyball).mp.
11. "leisure time physical activity".mp
12. 7 or 8 or 9 or 10 or 11
13. 6 and 12
14. life style/ or sedentary lifestyle/
15. (physical adj3 inactive*).mp. [mp = title, abstract, original title, name of substance word, subject heading word, keyword heading word, protocol supplementary concept word, rare disease supplementary concept word, unique identifier, synonyms]
16. physical inactiv*.mp. [mp = title, abstract, original title, name of substance word, subject heading word, keyword heading word, protocol supplementary concept word, rare disease supplementary concept word, unique identifier, synonyms]
17. (((sedentary or inactive or sitting) adj3 (time or behavio?r or life style or lifestyle)) or "screen time").mp. [mp = title, abstract, original title, name of substance word, subject heading word, keyword heading word, protocol supplementary concept word, rare disease supplementary concept word, unique identifier, synonyms]
18. 14 or 15 or 16 or 17
19. 6 and 18
20. 13 or 19
21. (physical activity assessment or physical activity measurement or energy expenditure or energy expenditure measurement or self report* physical activity or physical activity questionnaire* or physical activity log* or physical activity record* or physical activity recall or physical activity adult questionnaire* or PAAQ or international physical activity questionnaire* or IPAQ or Global physical activity questionnaire or GPAQ or Minnesota Leisure time Physical Activity Questionnaire).mp. [mp = title, abstract, original title, name of substance word, subject heading word, keyword heading word, protocol supplementary concept word, rare disease supplementary concept word, unique identifier, synonyms]
22. exp Motor Activity/
23. monitoring, physiologic/ or exp actigraphy/ or exp monitoring, ambulatory/
24. exp accelerometry/ or exp actigraphy/
25. (pedometer or omron pedometer or fitbit or jawbone or nike or activity tracker* or activity monitor or accelerometer* or wrist worn accelerometer or motion sensor* or activity monitor or inclinometer* or activPal or CSA monitor or heart rate monitor* or HRM or multi sensor system or MSS or actiheart or sensewear armband or zephyr bioharness or actical or actigraph*).mp. [mp = title, abstract, original title, name of substance word, subject heading word, keyword heading word, protocol supplementary concept word, rare disease supplementary concept word, unique identifier, synonyms]
26. 21 or 22 or 23 or 24 or 25
27. 20 and 26

### 2.4 Study selection

We identified 5,818 citations across eight databases, and exported them into Covidence (Victoria, Australia). Duplicates were removed resulting in 3,696 titles/abstracts for screening at Level 1. Two authors (BM, RA) independently reviewed all titles. At Level 2, BM, RA reviewed full text of all identified studies. If there was discordance at Level 1 or 2, a third author (MCA) reviewed and decided the final outcome. In instances where there were multiple publications from the same study, we included only one citation with baseline measures on sedentary time and physical activity.

### 2.5 Identification of further studies and additional data

We conducted a forward and backward (reference list) citation search for studies included at Level 2. We also identified five recent reviews relevant to our research focus and reviewed their reference lists [25–29].

### 2.6 Data collection process and data items

To create a physical activity profile for our population, we developed a standardized data extraction form. Two reviewers (BM and RA) independently conducted data extraction including study author; year of publication; study setting/location; study design; sampling method; participant demographics; sedentary time and physical activity outcome measures; monitor characteristics (make, model); data processing decisions (epoch length, wear time, calculation of non-wear time, cut-points applied to categorize sedentary time and physical activity intensity) etc. We emailed authors of five studies to seek further clarification regarding the cut-points applied, unit of reporting, break down of sample by ethnicity and gender, and reported levels of physical activity.

### 2.7 Summary measures and synthesis of results

Our primary outcomes were sedentary time and various intensity of physical activity. Sedentary time was defined as time spent for any duration (e.g., minutes per day) or in any context (e.g., at school or work) in sedentary behaviors. Sedentary behavior was defined as any waking behavior characterized by an energy expenditure ≤1.5 metabolic equivalents (METs), while in a sitting, reclining or lying posture [30]. Physical activity was defined as any bodily movement produced by skeletal muscles that results in energy expenditure [31]. A ≥10-minute bout of MVPA was defined as sustained MVPA bout accumulated in consecutive 10 minutes or more with one or two minutes of interruption. Sedentary time and physical activity outcomes were reported either as mean (SD) or median (IQR) minutes. We converted heterogeneous units of measurement, into ones reported most often. Due to inconsistencies in the way sedentary time and measures of physical activity were measured, defined and reported, we synthesized and presented this data narratively rather than a meta-analysis.

### 2.8 Quality assessment

To determine study quality, we used the Strengthening the Reporting of Observational Studies in Epidemiology (STROBE) guidelines [32], based on 22 items related to title, abstract, introduction, methods, results, and discussion sections of articles. We assessed the reporting quality of two RCTs with the CONSORT checklist [33].

We evaluated methodological quality of studies using the Newcastle Ottawa Quality Assessment Scale (NOS) [34].NOS has primarily been developed to assess quality of case-control or cohort studies. We adapted the NOS to make it more suitable for the assessment of quality of cross-sectional studies. We replaced/adapted irrelevant items and added 11 items on the completeness of accelerometer reporting (see S1 File Adapted NOS). Studies that compared differences in sedentary time and physical activity between South Asians and other ethnic group/s had two additional questions related to adjustment for important factors. Maximum points a study with or without a comparison group could achieve was 27 and 25 points respectively. Studies with a comparison group were classified as poor if they scored between 1–12 points, fair (13–19 points), or good (20–27 points). For studies without comparison groups, possible ratings were poor (1–11), fair (12–18), and good (19–25 points). Two authors (BM and RA) independently rated all studies.

## 3. Results

### 3.1 Selected studies

**Fig 1:**
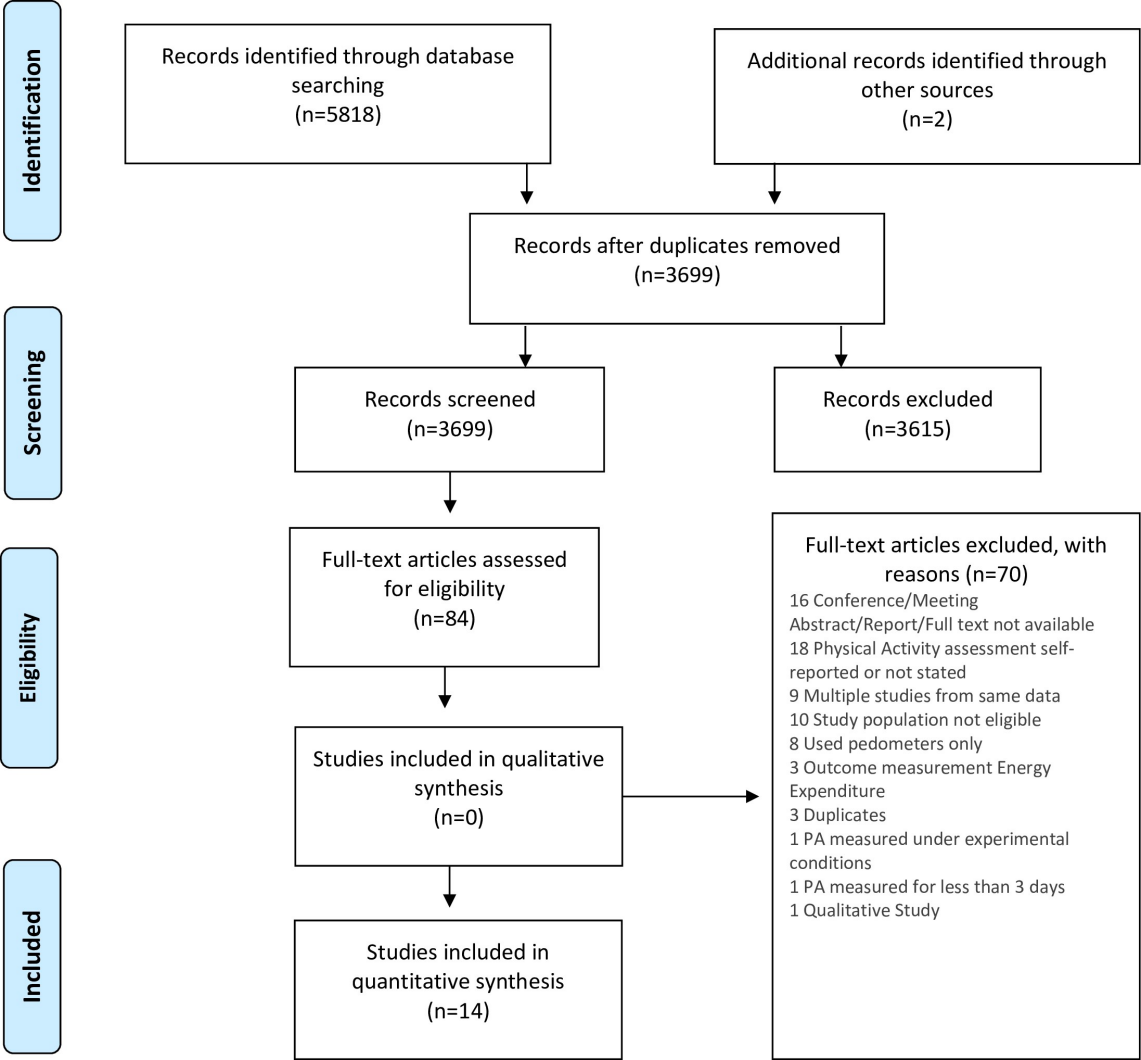
Flow diagram of studies considered for inclusion in the systematic review.

Our search identified 5,818 articles. After removing duplicates, 3,699 articles were reviewed for relevancy, with 84 full text articles assessed for eligibility. Seventy of these were considered irrelevant and excluded. Reasons for exclusion are outlined in Fig 1. We included twelve studies. Two studies reported on the same dataset, but one study reported sedentary time, and the other reported physical activity [35,36]. Backward and forward citation search of the selected studies revealed two more relevant studies thus leading to a total of 14 studies that were included in the final review (Fig 1).

### 3.2 Study characteristics

Of the 14 studies, six were conducted in United Kingdom (UK) [37–42], two in the United States (USA) [37–44], and one each in Canada [45], Norway [45,46], Netherlands [36], Singapore [47], India [48] and Bangladesh [49] (Table 1). Thirteen out of 14 studies used an ActiGraph (Pensacola, Florida, USA) with two using a Sensewear Armband (BodyMedia, Inc., Pittsburgh, PA) [41] and a pedometer [42] along with the ActiGraph. One study applied an Actiheart (CamNtech Ltd, Papworth, UK) [35,36]. Majority of the studies (n = 11) were cross-sectional

**Table 1:** Study and sample population characteristics included in the review.

Study Authors	Study Location	Study Design	Sample Population	Sample size (N)	Mean/Median Age (Yrs., SD/IQR)	Mean BMI (kg/m2), (SD), Classification % (Cut-Points)	Baseline Sedentary Time (ST)/Physical Activity (PA) Measures
Andersen, E. et al., 2011	Oslo, Norway	Cross-sectional	SA (Pakistani) men only (25–60 yrs.)	150	37.3 (7.7)	27.2 (3.6); (International Diabetes Federation)	Total PA (counts/min); Mean Inactive time (hours/day); Moderate intensity PA; Vigorous intensity PA; MVPA (min/day);
Castaneda-Gameros, D. et al., 2018	Birmingham, UK	Cross-sectional	SA; African Caribbean; & Other–(Arabs, White Irish) women only.	Total: 60	70.8 (8.1)	29.4 (4.8); (WHO BMI cut-points for Asians)	Mean ST (min/day); Low Light PA (min/day); High Light PA (min/day); MVPA (min/day)
				SA: 25			
				African/Caribbean: 23			
				Other:12			
Curry, W.B. et al., 2014	Wales, UK	Cross-sectional	SA (Pakistani, Bangladeshi) women only (18–72 yrs.)	167	46.3 (15.1)	27.8 (5.5)	Mean ST (min/day); Mean LPA (min/day); MVPA (min/day);
						(WHO BMI cut-points for Asians)	
Emadian, A. et al., 2017	London, UK	Cross-sectional	SA men only (18–65 yrs.)	63	44.8 (9.9)	28.1 (4.2)	Mean ST (min/day); Moderate intensity PA; Vigorous intensity PA; MVPA (min/week)
Iliodromiti, S. et al., 2016	Scotland, UK	Cross-sectional	SA; European.	Total: 364	Median: SA: 49 (42.0, 55.0)	SA: 27.1 (4.6)	Median MVPA (min/week); Bouted MVPA (min/week)
				(Valid data on 311).		European: 26.3 (4.4)	
					Europeans: 49 (44.0, 55.0)		
				SA:148 (73 women; 75 men)			
				European:163 (80 women; 83 men).			
Kandula, N.R. et al., 2015	Chicago, USA	RCT	SA (Indian, Pakistani)	Total: 63 (40 women, 23 men).	50 (8)	30 kg/m2 (5.0)	Mean ST (min/week); MVPA (min/week); Bouted MVPA (min/week).
Kandula, N. R. et al., 2016	Chicago, USA	Single arm Before-After	SA (Indian, Pakistani) women only	30	40 (5)	29.5 (2.5)	Mean Bouted MVPA (min/week).
Mumu, S.J. et al., 2017	Thakurgaon District and Dhaka, Bangladesh	Cross-sectional	SA	Total: 162	35 (9)	Not reported	CPM/day; Mean Steps/day; Mean ST (min/day); Light PA (min/day); MVPA (min/day).
			(Bangladeshi)				
				Rural: 97; Urban: 65 (88 women, 74 men).			
Mathews, E. et al., 2013	Trivandrum, India	Cross-sectional	SA (Indian) women only	47	46.4 (3.1)	Not reported	Mean Step count/day; Mean ST (min/week); Moderate intensity PA; Vigorous intensity PA; MVPA (min/week); Bouted MVPA (min/week);
Nicolaou, M. et al., 2016; Loyen, A. et al., 2017	Amsterdam, Netherlands	Cross-sectional	SA Surinamese; Dutch; African Surinamese; Turks; and Moroccan.	Nicolaou et al: Total: 462; Loyen et al: Total: 447 (ST).	Total sample: Median 49.0 (41.0–54.0);	Total Sample Median BMI: 26.1 (23.3–29.1);	Mean Sedentary Time (min/day); Light PA (min/week); Moderate and High Intensity PA (min/week);
						SA Women (Mean): 26.1 (4.7), SA Men: 26.3 (4.1)	
				SA Surinamese: 98 (55 women, 43 men); Dutch: 111 (76 women, 44 men); African Surinamese: 91 (42 women, 49 men); Turks: 88 (40 women, 48 men); Moroccan: 74 (37 women, 37 men).			
					SA Women (Mean): 45.5 (10)		
					SA Men (Mean): 48.9 (9.3)		

Pollard, T.M. et al., 2012	Newcastle, UK	Primarily Qualitative	SA (Pakistani) women only	22	40.3 (10.8)	28.4 (6.2)	CPM per/day; Mean ST (min/day); Mean Step count/day; MVPA (min/day).
Sumner, J. et al., 2018	Singapore	Cross-sectional	SA (Indian); Chinese; Malay.	Total: 713 (417 women, 296 men); SA: 100; Chinese: 492; Malay:121	Total Sample: 47.8 (14.6);	Healthy (< 23 kg/m2) Total: 298 (42%); Indian: 20 (20%); Chinese: 243 (49%); Malay: 35 (29%);	Mean Step count/day; Percent categorized as sedentary based on step count; % of time spent in each cadence band from 0 steps/min (non-movement) to ≥120 steps/min (brisk walking or faster); Mean peak 1^-^min, 30 min, 60 min cadence minutes (SD); amount of time (minutes) and proportion of time (%) accumulated in previously defined cadence bands; Proportion of participants accumulating 30-min/day at ≥100 steps/min
					SA: 46.5 (14.4): Chinese: 49.1 (14.2); Malay: 43.9 (15.8)		
						Overweight (23–27.4 kg/m2) Total: 280 (39%); Indian: 52 (52%);	
						Chinese: 180 (37%); Malay: 48 (40%)	
						Obese (≥27.5 kg/m2) Total: 134 (19%) Indian: 28 (28%); Chinese: 68 (14%); Malay: 38 (31%)	
Tong, C.E et al., 2017	Vancouver, Canada	Cross-sectional	SA (Indian, Pakistani, Fijian); Chinese.	Total: 49; (37 women, 12 men).	Total Sample: 73.8 (6.1)	Total Sample Mean: 26.3 (4.5)	Mean Step count/day; Percent (%) Mean of Sedentary Time; Light PA, Lifestyle PA and Moderate and Vigorous activity as a mean percent of total daily activity time.
				SA: 20; Chinese:29			
Yates, T. et al., 2015	Leicestershire, UK,	Cross-sectional	SA; White.	Total: 3,086; Walking Away 561; (1137 women, 1949 men)	SA: 58 (9)	Walking Away:	Median Steps/day; Median MVPA (min/day). Bouted MVPA min/week;
					White: 64 (8)		
						SA 29.7 (4.6); White 32 (5.0)	
				SA: 243 (83 women, 160 men)		Let’s Prevent:	
						SA 30.1 (5.9); White 32.2(5.5)	
				White: 2,843 (1054 women, 1789 men).			

SA = South Asian

### 3.3 Study quality

Overall, study quality was low with 21% (n = 3) classified as ‘poor’, 71% (n = 10) as ‘fair’ and 7% (n = 1) as ‘good’. Studies lost points on the sample not being representative, missing information on sample calculation/justification, and response rate. Studies scored higher on accelerometer-related items with 71% (n = 10) fulfilling basic requirements.

### 3.4 Participants’ characteristics

A total of 5,439 participants (sample size range 22–3,086) were enrolled in 14 studies; 53% were women. Total number of South Asians recruited across 14 studies was 1,338 (range 22–243): Six out of 14 studies included other ethnic groups. Mean age of the participants ranged from 35 (9) to 73.8 (6.05) years, and mean BMI ranged from 27.1 (4.6) kg/m^2^ to 32.0 (5.0) kg/m^2^ (Table 1). Due to unstandardized and inconsistent reporting of education, income, time since immigration and employment status, it was not possible to present conclusive results regarding these socio-demographic variables.

### 3.5 Accelerometer data collection and processing decisions

Majority of studies required a seven-day monitoring period (n = 12), with an accelerometer-wear inclusion criterion of ≥10 hours (600 min/day) (n = 11), with at least 4 valid days (n = 6), and with epoch length of devices set at 60 seconds (n = 10). Five studies reported compliance rate for accelerometers (range 51–91%) (Table 2).

**Table 2:** Accelerometer related data collection and processing decisions.

Author/ Year	Accelero-meter Model	Days of Data Collection	Epoch length (seconds)	Criteria for defining non-wear time	Wear time requirement	Min number of valid days required	Mean wear days (SD) Mean time per day (SD)	Hours of data collection (e.g. waking hours only)	Accelero-meter compliance strategies	Accelerometer Compliance
Andersen, E. et al., 2011	MTI ActiGraph, Model 7164	7	60	60 min of continuous zero counts with exception of two interruptions	480 min/day	2	Wear Days: 6.3 (1.8);	Waking hours only except while swimming or bathing	Not specified	Not specified
							Wear Time: 13.5 hrs/day (1.5).			
Castaneda-Gameros, D. et al., 2018	ActiGraph GT3X	7	60	90 min of consecutive zero counts, with an allowance of 2 minutes of nonzero counts provided there were 30-minute consecutive zero count windows up and downstream	600 min/day (10 hrs/day)	3 (including one weekend day)	Wear Days: 5.7 (1.3);	Waking hours only except while swimming or bathing	Text messages; phone calls; pictorial log.	Low (79%). Compliance rates of non-English speakers lower than for women with English literacy (63% vs. 88%)
							Wear Time: 778.3 min/day (71.9)			
Curry, W.B. et al., 2014	ActiGraph GT1M and GT3X	7	60	60 min of consecutive zeros	600 min/day (10 hrs/day)	3 (including one weekend day)	Not specified	Waking hours only except while swimming or bathing	Phone calls or text messages; pictorial diary.	(90.9%)
Emadian, A. et al., 2017	ActiGraph GT3X	7	60	> 60 min of consecutive zero activity counts	600 min/day (10 hrs/day)	4	Not specified	Waking hours only except while swimming or bathing	Not specified	Not specified
Iliodromiti, S. et al., 2016	ActiGraph G3TX+ Actitrainer	7	60	60 min of consecutive zeros	600 min/day (10 hrs/day)	4	Not specified	Waking hours only except while swimming or bathing	Not specified	Not specified
Kandula,N.R. et al., 2015	ActiGraph 7164	7	Not specified	Not specified	Not specified	Not specified	Wear time: Intervention Group: 6062 (1376) min/wk; Control Group: 5970 min/wk (1268)	Not specified	Reminder calls	Not specified
Kandula,N. R. et al., 2016	ActiGraph 7164	7	Not specified	Not specified	600 min/day (10 hrs/day)	Not specified	Not specified	Not specified	Not specified	Not specified
Mumu, S.J. et al., 2017	ActiGraph GT3X	7	60	Not specified	600 min/day (10 hrs/day)	3 (including one weekend day)	Not specified	Waking hours only except during water based activities.	Not specified	Not specified
Mathews, E et al., 2013	ActiGraph GT3X	7	60	60 min of consecutive zeros	600 min/day (10 hrs/day)	4	Not specified	Waking hours only.	Daily Reminders	51.1%
Nicolaou, M. et al., 2016; Loyen, A. et al., 2017	Actiheart V4 CamN Tech Ltd UK	5 (at least one weekend day)	NA	Not specified	To be worn continuously– 24 hrs	4	Not specified	Worn continuously during the measurement period, including during showering, water sports and sleeping	Not specified	Not specified
Pollard, T.M. et al., 2012	ActiGraph GT1M and Sensewear Armband Pro 3	4 (2 weekend days)	Not specified	For Actigraph: 30 min of consecutive zeros	For Actigraph: 600 min/day (10 hrs/day)	For ActiGraph 3 days; Sensewear: 1 day	Not specified	Actigraph: Waking hours only. Sensewear Armband: at all times except when bathing or swimming:	Information Sheet; diary; visits by staff asking participants to recall activities and their intensities for previous day.	60%
Sumner, J. et al., 2018	ActiGraph GT3X+	7	60	Not specified	600 min/day (10 hrs/day)	4	Wear time: 907.5 (116.8) min/day Chinese: 899.4 (112.5) min/day Malay:939.4 (131.5) min/day Indian: 908.9 (112.9) min/day	To be worn continuously except bathing or swimming	Not specified	Not Specified
Tong, C.E et al., 2017	ActiGraph (Model not stated)	7	60	>120 minutes of consecutive zeros, allowing for 1–2 minutes of counts 0–100	600 min/day (10 hrs/day)	6	Wear Time: 14 hrs/day (839 min/day; Range: 11.4–16.8 hours/day).	Waking hours only- except when swimming or bathing	Translated take-home booklets with written and pictorial instructions; logs	Not specified
Yates, T. et al., 2015	ActiGraph GT3X	7	Activity recorded in 15 sec epochs and then reintegrated into 60 sec epochs	60 min of consecutive zeros	600 min/day (10 hrs/day)	4	Not specified	Waking hours	Not specified	Not Specified

### 3.6 Accelerometer-based sedentary time and physical activity outcome measures

#### 3.6.1 Sedentary time

**Fig 2:**
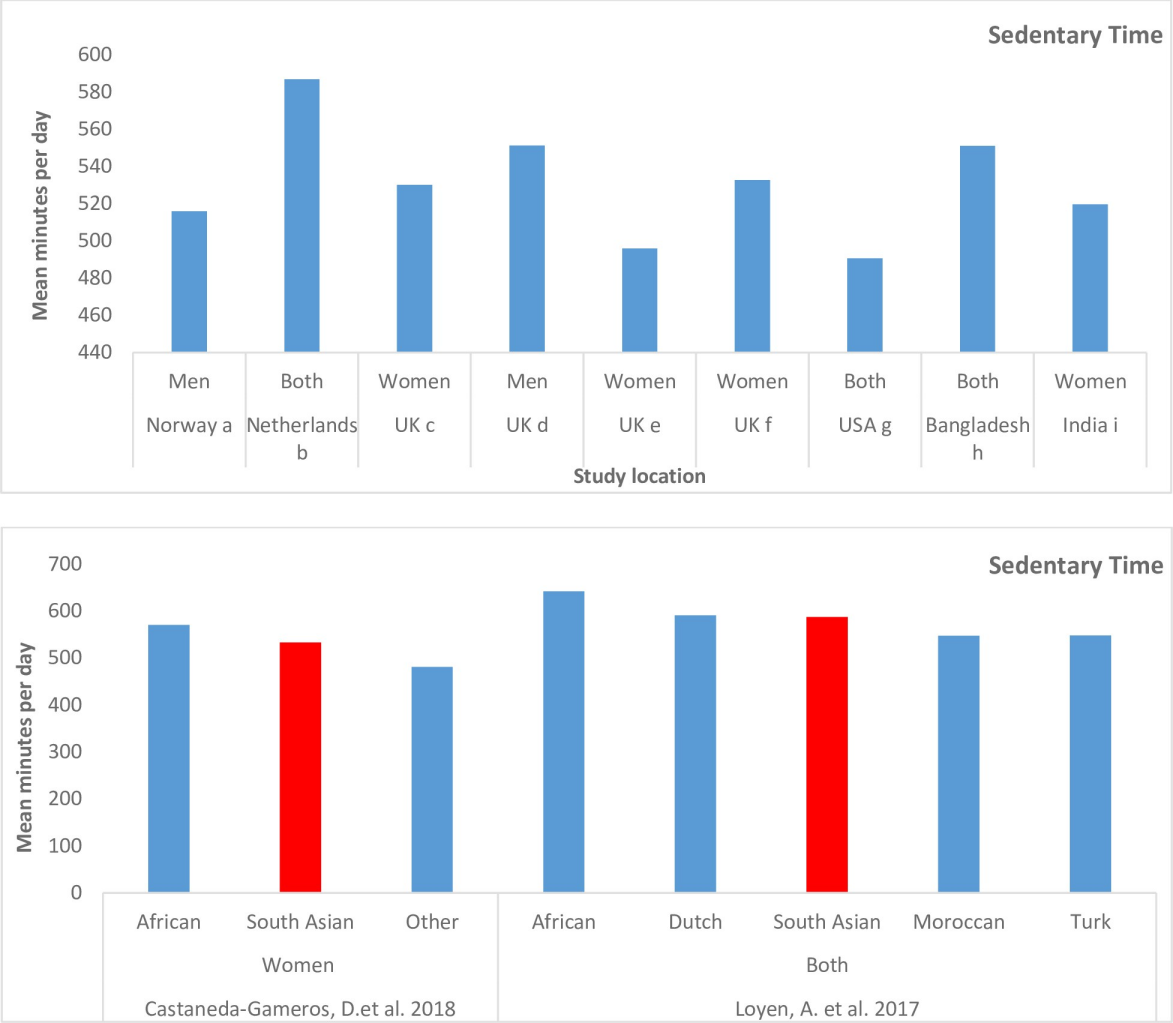
a) Prevalence of sedentary time among South Asians by study location. ^a^Andersen, E. et al., 2011; ^b^Loyen, A. et al., 2017; ^c^Curry, W.B. et al., 2014; ^d^Emadian, A. et al., 2017; ^e^Pollard, T.M. et al., 2012; ^f^Castaneda-Gameros, D. et al., 2018; ^g^Kandula, N.R. et al., 2015; ^h^Mumu, S.J. et al., 2017; ^i^Mathews, E. et al., 2013. b) Mean minutes per day of sedentary time by ethnic groups.

Eleven of 14 studies reported sedentary time. Within South Asian sample, sedentary time ranged from 482 (98) min/day [43] to 587 min/day (Fig 2A) [35].

Four studies compared sedentary time of the South Asian cohort with other ethnic cohorts. Sumner et al. performed analysis of covariance and pairwise comparisons to test for differences in continuous stepping parameters by ethnicity and differences in mean peak cadence by step categories. The proportion of Malays, Indians, and Chinese achieving < 5000 daily steps (considered sedentary) was 26%, 23% and 14%, respectively (p < 0.01).[47].

Compared with a Chinese sample, the percent mean sedentary time in another study, was slightly greater in South Asians: 67.9% (9.5) vs 66.3% (7.9) [50].

After adjusting for age and wear time, Castaneda-Gameros, D. et al., reported significantly high min/day of sedentary time in the African Caribbean sample 570.1(70.6) followed by South Asians 532.8 (102.4) and other 481.2 (53.2) (p < 0.04).

Loyen et al., investigated sedentary time min/day in five ethnic groups The highest sedentary time was reported for African Surinamese (621min/day), followed by South Asians (580 min/day) (Fig 2B). Once adjusted for gender and age, differences among ethnic groups were not significant.

### 3.6.2 Light physical activity

Five out of 14 studies reported light physical activity (Table 3).

Within the South Asian sample, mean light physical activity range was 211.7 (67. 3) [49] to 574.4 (227) min/day [36]. Curry et al., reported significant differences in mean min/day of light physical activity performed over weekdays vs weekends in women born in UK 316.5 (106.7) vs those born in Pakistan 253.4 (95.1) or Bangladesh 288.9 (113.8), in women under 65 years of age and those belonging to under-weight or normal weight category (Table 3). Three out of five studies compared light physical activity levels of South Asians with other ethnic groups. Castaneda-Gameros, D. et al., found that low light physical activity and high light physical activity (adjusted for age and accelerometer wear time) was significantly different in the African Caribbean 168.9 (60.0) (p = 0.01) and 16.8 (11.7) (p < 0.04). compared to other (Arabs and White Irish) and South Asian groups.

Nicolaou et al., reported second highest mean min/day of light physical activity for South Asian women, [574 min/day (227.7)] compared with women in other ethnic groups. In men, the highest number of mean minutes of light physical activity were observed in Moroccans [641 (247) min/day], while South Asian men were second in last [518 (235) min/day] just above Dutch [36]. Compared with South Asian men, South Asian women had higher levels of light physical activity. Tong et al., reported slightly higher mean percent of the total daily light physical activity in Chinese compared with South Asians [24.4% (6.8) vs 22.7% (6.5)] [50].

**Table 3:** Prevalence of sedentary time (ST) and various intensity physical activities (PA).

Author/Year	SA Sample with valid accelerometer data	ST and PA intensity Cut points (counts per minutes)	Sedentary time	Step Count	Light PA	Moderate PA	Vigorous PA	MVPA	MVPA in 10 min Bouts	Within- study comparison with other ethnic groups
Andersen, E. et al., 2011	SA: 142 (Men only)	Matthews: Sedentary ≤100	Mean min/day 516 (96)					Mean min/day: 32.3 (20.8)		
		Freedson: Light: 101–1951,								
		Moderate: 1952–5724,								
		Vigorous: 5725–9497; Very Vigorous: >9497								
Castaneda-Gameros, D. et al., 2018	Total: 60	Sedentary: <100; Copeland: Low-Light PA: 100–1,040; High-Light PA: 1,041–1,951; Freedson: MVPA ≥1,952	Mean min/day SA: 532.8 (102.4) African/ Caribbean: 570.1 (70.6) Other: 481.2 (53.2)		Mean daily min/day SA: LLPA: 207.9 (69.6); HLPA: 30.2 (19.2);			Mean min/day SA: 20.0 (21.4)		SA’s second highest sedentary group after African Caribbean; second highest mean minutes of LLPA and HLPA (higher than African Caribbean); and MVPA after Other (Arab and Irish)
	SA: 25									
Curry, W.B. et al., 2014	SA: 140 (Women only)	Matthews: Sedentary <99; Freedson: Light 100–1951; Moderate: 1952–5724; Vigorous 5725–9498; Very Vigorous 9499- ∞/min	Mean min/day 530.2 (81.8)		Born in UK: 316.5 (106.7); Born in Bangladesh: 288.9 (113.83); Born in Pakistan; 253.4 (95.1)			Mean min/day: 34.7 (21.5); Weekdays: 36.12 (12.5) Weekends: 27.4 (14.2)		
Emadian, A. et al., 2017	SA: 54 (Men only)	Freedson: Sedentary 0–99; Light 100–1951; Moderate: 1952–5724 Vigorous: 5725–9498; Very Vigorous: >9499	Mean min/day: 551.4 (95.0)					Mean min/day: 42.7 (26.7)		
Iliodromiti, S. et al. 2,016	Total: 311; SA:148	Freedson: MVPA: ≥1952						Median min/day (IQR) SA: 23.8 (11.41, 42.45)	Median min/day 2.85 (0, 13.1)	Europeans median MVPA and bouted MVPA higher than SA’s (45.28 (28.2, 70.71) and 5.71 (15.85, 33.57) respectively.
Kandula,N.R. et al., 2015	Not specified	Not Specified	Mean min/day: 482 (98.4) Intervention Group; 499.3 (110.7) Control Group; 490.6 (104.6)					Mean min/day baseline: Intervention Group: 27.3 (26.85); Control Group: 20.1 (20.1)	Mean min/day Intervention Group: 8.0 (16.3); Control Group: 6.3 (15)	
Kandula,N. R. et al., 2016	Not specified	Not specified							Mean min/day 6 (14.9)	
Mumu, S.J. et al., 2017	SA: 155	Atkin and Freedson et al: Sedentary: <100; Light: <1952; Moderate: 1952–5724; Vigorous >5724	Mean min/day: 551.2 (83.0) Rural: 554.3 (81.4); Urban: 546.4 (85.9);	Mean Steps per day 9998 (3936) Rural: 8658 (2788); Urban: 12,063 (4534)	Mean daily min/day: 211.7 (67. 4); Urban = 205.8 (59.1); Rural = 220.8 (78.1).			Mean min/day: 57.9 (30.4) Urban = 51.1 (25.6) Rural = 68.6 (34.2); Diff between rural and urban sig (p-value = 0.001)		
Mathews, E et al., 2013	SA: 24 (Women only)	Freedson’s; Sedentary <100; MVPA: ≥1952	Mean min/day: 519.7 (115.1)					Mean min/day: 16.7 (10.9)	Mean min/day 5.7 (7.8)	
Nicolaou, M. et al., 2016; Loyen, A. et al. 2017	SA: 98	Cut points based on Dutch PA norm (NNGB); Sedentary time: *waking* time spent in activities *<*1.5 MET; Light Intensity: all activity between 1.5 < 4.0 METs for <55 yrs old; and 1.5 < 3.0 METs for > 55 yrs old. Moderate- and high intensity activities: for < 55 years between 4.0 and 6.5 METs; adults aged ≥ 55 yrs 3.0 and 5.0 METs, respectively.	Mean min/day: 587		Mean daily min/day: SA women = 574.4 (227.7) /day, SA Men = 518 (235.2);			Mean min/day SA women: 41.1 (71); SA Men: 36.6 (43.7)		SAs had the second highest (adjusted) mean min of ST after Africans. SA women had second highest LPA after Moroccan women; SA men had the fourth lowest levels of LPA–just above the Dutch 429 (196). Of the five ethnic groups, SA men had lowest MVPA and SA women had second lowest mean min of MVPA
Pollard, T.M. et al., 2012	SA: 14 (women only)^a^	Freedson: Sedentary <100; MVPA: ≥1952	Mean min/day: 496 (64)	Mean Steps per day: 5368 (2073)				Mean min/day 17 (8)^a^		
Sumner, J. et al., 2018	Total:713 SA: 100	Tudor-Locke C, Bassett DJ: Sedentary: < 5000 steps; Low activity: 5000–7499 steps; Somewhat active: 7500–9999 steps; Active: ≥ 10,000 steps/day	23% SAs sedentary	SA: 7083.9 (2839.0)		SA: 15% achieving 30 min of moderate intensity PA through steps (≥100 steps/min)				Proportion of SAs classified as sedentary second highest after Malays (26%). SA’s had lowest mean daily steps vs Malays 7140 (3073.3) and Chinese 7745 (2774.4); proportion of SA’s achieving 30 min of moderate PA significantly higher in Chinese (32%) vs SAs (15%) and Malays (16%).
Tong, C.E et al., 2017	Total: 46 SA: 17	Eslinger & Copeland: Sedentary: 0–50 CPM; Light: 100–759: Lifestyle: 760–1951: Freedson: MVPA: ≥ 1952–9498; Very Vigorous: ≥ 9499	Percent mean sedentary time of SA’s: (67.9% (9.5)	Mean daily Steps SA (7196.40 (3854.49), Min/Max = 1939.1/16290.9	Mean % of daily LPA SA: 22.7% (6.50); Lifestyle PA SA: 6.86 (2.34);	Moderate activity as a mean percent of total daily activity time, SA: 2.81 (2.35);	Vigorous activity as a mean percent of total daily activity time, SA: 0.03 (0.02)			% mean sedentary time slightly greater in South Asians vs Chinese; Step count greater in Chinese (8291.05 (3569.78); % mean of daily LPA and Lifestyle PA higher in Chinese: Light PA: 24.40 (6.75) Lifestyle PA: 6.18 (2.4); % mean of total daily moderate activity time slightly higher in Chinese 3.13 (2.68)
Yates, T. et al., 2015	Total: Walking Away: 605; SA: 44	Freedson: MVPA: ≥1952		Median Steps/day SA: 5992 (4351, 8422)				Median min/day SA: 18.0 (7.5, 36.1)	Median min/day SA: 2.5 (0, 19.1)	Median steps higher in White: 6157 (4372, 8171); Median min/day of MVPA and bouted MVPA higher in Whites: 21.5 (10.5, 41.6) and 4.0 (0, 14.45) respectively

^a^ Only ActiGraph data reported

SA = South Asian

#### 3.6.3 Moderate to Vigorous Physical Activity (MVPA)

**Fig 3:**
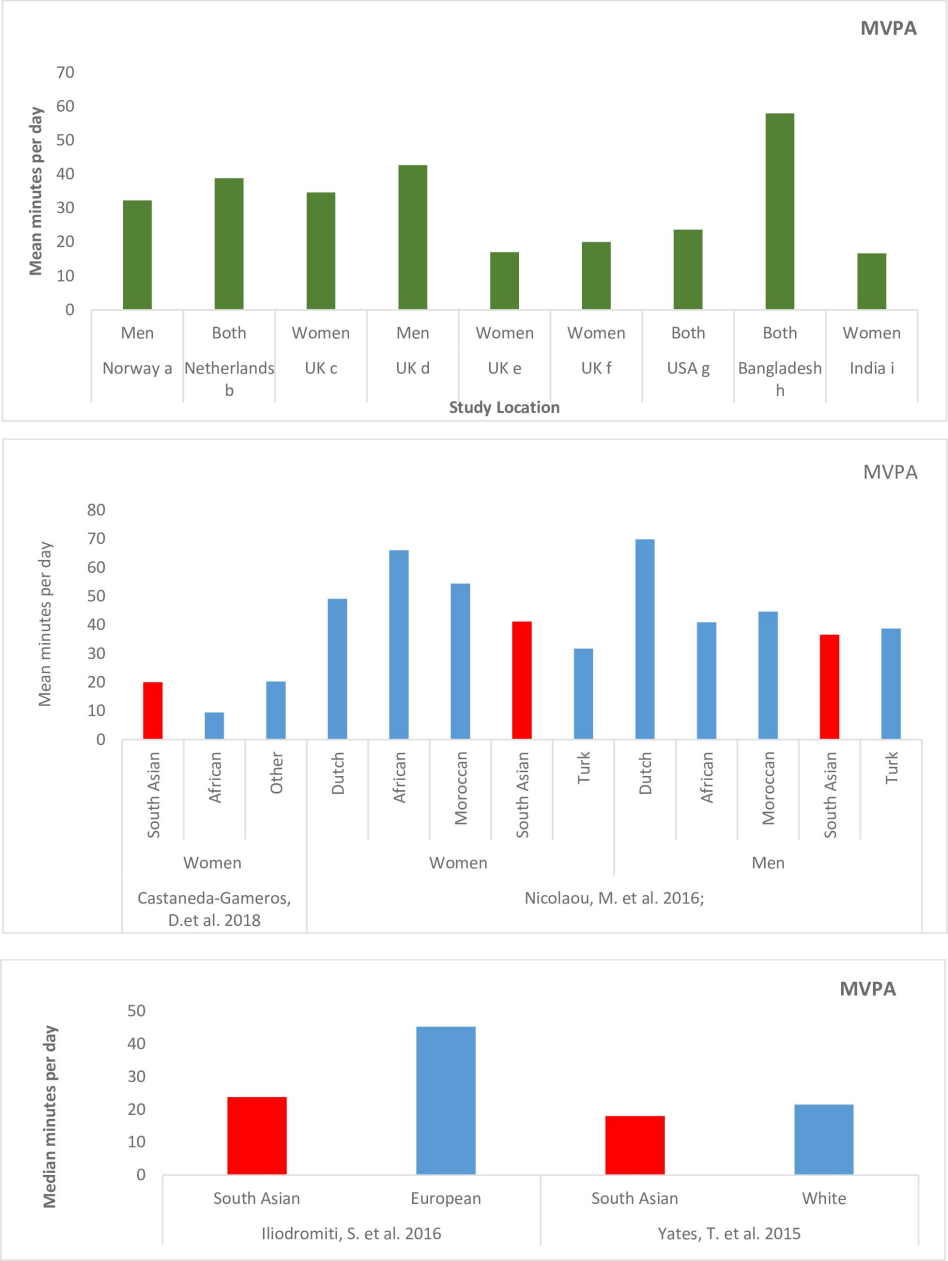
a) Prevalence of moderate to vigorous physical activity among South Asians by study location. ^a^Andersen, E. et al., 2011; ^b^Loyen, A. et al., 2017; ^c^Curry, W.B. et al., 2014; ^d^Emadian, A. et al., 2017; ^e^Pollard, T.M. et al., 2012; ^f^Castaneda-Gameros, D. et al., 2018; ^g^Kandula, N.R. et al., 2015; ^h^Mumu, S.J. et al., 2017; ^i^Mathews, E. et al., 2013. c) Median minutes per day of MVPA by ethnic groups.

Mean min/day of MVPA for South Asian sample ranged from 16.7 min/day [48] to 57.9 (30.4) min/day [49] (Fig 3A) (Table 3).

Mumu et al reported significant differences in the MVPA for rural versus urban participants (51 min/day for rural and 69 min/day for the urban, p = 0.001). Illiodimitri et al., reported significantly higher median MVPA min/day for White, [45.3 (IQR 28.2, 70.7) min/day], compared with South Asians [(23.8 (IQR 11.41, 42.5P = 0.0001. Castaneda-Gameros D. et al., observed lowest levels of MVPA in African Caribbean group [9.5 (11.0) min/day)], followed by South Asians [20.0 (21.4) min/day] and others [20.3 (18.2)]. These differences were not statistically significant though. Yates et al., also observed slightly higher median minutes per day of MVPA in White [21.5 (IQR 10.5, 41.6)], compared with South Asians [18.0 (7.5, 36.1)]. Once adjusted for age, sex, social deprivation and accelerometer wear time, these differences were not significant [42]. Among women of various ethnic groups, Nicolaou et al., found lowest mean minutes of MVPA per day in Turkish women [31.7 (22.4)], followed by South Asian women [41.1 (71)]. Among men, South Asians had the lowest levels of MVPA [36.8 (43.7) min/day] (Fig 3B and 3C) [36].

### 3.7 Physical activity guidelines

In studies focusing on South Asians only, percent of the sample meeting physical activity guidelines of 150 min/week of MVPA ranged from 53% [38] to 61.1% [39]. The percentage range of participants meeting bouted MVPA guidelines ranged from 4.2% [46] to 35% [38]. All these studies reported considerably smaller proportion of South Asian sample meeting these requirements (15% Indian, 30% South Asian women (vs 43% Dutch women) and only 24% South Asian men (vs. 43% Dutch men) [36,47].

## 4. Discussion

To our knowledge, this is the first systematic review of studies using accelerometry to objectively measure sedentary time and physical activity in South Asian adults. Compared to a 2012 systematic review which identified only one study, we observed a 14-fold jump in the number of studies applying accelerometers to capture physical activity and sedentary time levels in South Asians [26]. Mean sedentary time among South Asians ranged from 482 to 587 min/day; light physical activity was between 211.7 to574.4 min/day; and mean MVPA ranged from 16.7 to 58 min/day. In most studies that compared these outcomes with other ethnic groups, South Asians consistently ranked relatively higher in sedentary time and light physical activity and lower in MVPA. Moreover, proportion of South Asians meeting physical activity guidelines of 150 minutes per week of MVPA was consistently lower compared with other ethnic groups.

We compared sedentary time and physical activity outcome measures with three population-level surveys that have applied an accelerometer for the assessment of these measures; Canadian Health Measures Survey 2007–09 (CHMS) [51], US-based National Health and Nutrition Examination Survey (NHANES 2003–04) [52], and the Swedish Attitude Behaviour and Change Study 2001–2002 [53]. While considerable heterogeneity among our studies limited our ability to identify any clear trends regarding sedentary time and physical activity levels, overall, the range for sedentary time was higher for South Asians when compared with NHANES and the Swedish survey but relatively lower when compared with the Canadian population-level survey. For light physical activity, reported levels for South Asian women were lower than those observed for American and Swedish women and closer to (but higher) than ones observed for Canadian women with the exception of one study reporting levels far surpassing all three national-level studies [36]. For MVPA, we observed significant variability between our studies and also when comparing against population-level surveys. Among studies that looked at MVPA in men only or provided MVPA prevalence by gender, the observed levels were comparable to ones reported for men by NHANES and the Swedish study but higher than the ones reported for Canadian men. Among women, the prevalence levels were closer to the high levels observed in the Swedish population. However, due to considerable heterogeneity, small sample sizes of the selected studies and different methodologies as well as different types of accelerometers and data processing decisions, these comparisons need to be interpreted with caution.

South Asians engaged in high levels of daily sedentary time (more than eight hours/day) (Fig 2A and 2B). With an established link between sedentary time and an increased risk of premature mortality and cardio-metabolic disease [54] it is imperative to reduce total sedentary time in high-risk populations like South Asians. When compared with data from NHANES, and CHMS, sedentary time range among South Asian adults is higher than one reported for the American population (464 min/day), [55] but falls within the range reported for the Canadian population (575 and 585 min/day for men and women respectively) [51]. Highest mean minutes of sedentary time (587 min/day) among the selected studies was reported by Loyen and colleagues who used an Actiheart. These monitors provide more accurate estimates of energy expenditure during lower intensity activities of daily living [56].

Previous studies have reported an increase in sedentary time and declining physical activity levels with age for both men and women, with some studies reporting higher sedentary time among older men than women [57,58]. While we did not see any clear patterns of sedentary time based on gender, Tong et al., reported a higher sedentary time for older Chinese and South Asian men compared with women. Higher sedentary time among older men (60 to 75 years old) is corroborated by data from Swedish study as well as NHANES, both of which reported higher sedentary time for older men compared to women [53,55]. Due to absence of similar data for South Asians from national surveys and given the small sample size of our studies, we could not draw any firm conclusion in our review. Nationally representative samples including South Asians of all age groups would enable a more effective comparison regarding how sedentary time is distributed by age and gender.

South Asians have been observed to lack knowledge regarding health risks of high sedentary time [59]. Some mixed-methods studies in our review reported an inability of the participants to comprehend, define or quantify their sedentary time [39] This may partly explain as to why sedentary time in South Asians is consistently higher than in other ethnic groups and underscores the need for further research on how sedentary time is perceived by South Asians as well as educating them on the detrimental health effects associated with long, uninterrupted sedentary behavior [60].One strategy that has been suggested to decrease sedentary time is to replace it with light intensity physical activities [61]. In fact, Buman et al., found reductions in cardiovascular disease risk biomarkers by simply substituting 30 min/day of sedentary time with an equal amount of light-intensity activity [62]. Consistent with previous research [63,64], some studies in our review found women engaged in greater light intensity physical activity than men [35,50]. Thus, future interventions directed at men may consider substituting sedentary time with light intensity physical activities. This has been suggested as a more realistic and practical first part of a stepped approach in increasing physical activity [65,66].

Despite majority of the studies having applied Freedson’s cut-points to classify MVPA, the reported MVPA range was wide, reflecting considerable heterogeneity and making it difficult to reach any definitive conclusions. For men, MVPA levels were closer to ones reported for American (31min/day) and Swedish (35 min/day) middle-aged adults and higher than those reported for Canadian men (26 min/day). For women, at least two out of four studies reported levels that exceeded the ones observed in the American (18 min/day), Swedish (32 min/day) and Canadian (21 min/day) women. Relative to women, men have been shown to accumulate higher levels of MPVA [51–53] However, no clear pattern regarding distribution of MVPA by gender emerged in our studies. Lowest MVPA levels (16.7 min/day) were observed in a sample of middle-aged, city-dwelling, married South Asian women in Bangladesh and highest (57.9 min/day), in a mixed sample of both urban-rural residents in India with MVPA being significantly higher in the rural residents (Fig 3A). Data from World Health Organization’s STEPS survey (focused on surveillance of non-communicable disease risk factors for the South-Asian countries) corroborates these urban-rural differences in inactivity rates [21]. Moreover, previous research have also reported higher overweight and obesity among urban South Asian women [67].

Within the studies that assessed MVPA levels among different ethnic groups, lower levels of MVPA were observed in South Asian men and women compared with other ethnic groups (Fig 3B and 3C). This is consistent with reports based on self-reported physical activity from Canada and United Kingdom [18,19,29,68]. However, objectively measured MVPA levels in South Asians in several of our studies appear to be within and at times even exceed the ranges observed in US, Swedish and Canadian populations. One factor contributing towards low levels of self-reported MVPA among ethnic populations could be that instead of capturing physical activity in all domains including occupation, majority of the self-report questionnaires mostly assess leisure time physical activity and levels for this type of activity are usually observed to be on the lower end for ethnic, specifically immigrant populations [69].

Current Canadian guidelines for physical activity require adults to accumulate at least 150 minutes of MVPA per week (or 30 minutes per day for at least 5 days a week) in bouts of 10 minutes or more [70]. CHMS 2014–15 reports only 18% of Canadian adults meeting these recommendations. While the percent of South Asians meeting physical activity guidelines in our selected studies was overall low, and even lower for bouted MVPA, compared with national-level data, at least two studies reported a higher proportion of South Asian participants adhering to guidelines [24% [39] and 35% [38]]. However, these findings need to be replicated in a larger sample.

Physical activity guidelines are based on evidence that has been derived from studies largely conducted on white, European populations [71] and may not necessarily apply to other ethnic groups like South Asians who, compared to White, with an equivalent level of physical activity, are still at an increased risk of cardio metabolic diseases, [10] and have lower levels of cardiorespiratory fitness and reduced ability to oxidize fat during exercise [72]. In fact, a 2013 study from UK has concluded that men of South Asian ethnic origin may need to undertake about 200–250 minutes per week of physical activity to obtain equivalent benefits [73]. This differential risk profile warrants the need for physical activity guidelines to be ethnic-specific.

Recruitment of ethnic minorities and socially disadvantaged populations in research has long been recognized as a challenging task [74]. This often leads to researchers opting for non-random samples that are relatively easier and less resource intensive. This may explain why majority of the studies in our review had convenience samples which may have introduced selection bias by skewing participation towards people who are more health conscious and perhaps more physically active. This may also explain the unexpected pattern of majority of the studies showing physical activity prevalence levels among South Asians only slightly lower to or exceeding those reported in nationally representative samples.

Apart from methodological issues, a more consistent and standardized reporting of socio-economic variables such as education, income, time since immigration, and type of employment would have provided useful information regarding the context in which most of the sedentary behaviour or physical activity occurred. From a public health perspective, this information is highly relevant as a first step towards designing effective, targeted physical activity interventions to increase physical activity in South Asians.

### 4.1 Strengths and limitations

This review is an important update on a previous review published in 2012 on levels of sedentary behavior and physical activity levels among South Asian women. Considering the well-recognized advantages of objective measurement of sedentary time and physical activity, we limited our focus to studies that applied an accelerometer to capture these measures. Our search strategy was comprehensive targeting seven databases with no time limits on the year of publication, or on language. Despite considerable heterogeneity among the selected studies limiting our ability to draw any firm conclusions, this review, nevertheless provides a credible physical activity profile of South Asians based on objective assessment. We were able to make comparisons with three population based surveys and develop a better understanding of where South Asians stand with respect to other populations. This review also identifies avenues for future research which can fill up some of the gaps that were highlighted above. Apart from these strengths, we would also like to highlight several limitations in the present review. Since we only searched published studies, a possibility of publication bias exists. We did not assess risk of bias in the selected studies as our primary focus was on baseline measures of sedentary time and physical activity only to enable us to create a profile of activity levels of South Asians. Considerable heterogeneity among studies limited our ability to conduct a meta-analysis or reach any definitive conclusions.

Majority of the studies had small sample sizes which were un-representative and therefore limited generalizability of the results. Information on certain socio-demographics (known to influence physical activity and sedentary behavior) like income, employment, education etc was also limited and inconsistent thereby curtailing our ability to draw any definitive conclusions regarding their association with our primary outcomes. Additionally, while comparisons with population level surveys were done to create a comprehensive activity profile for South Asians, one of these surveys- the CHMS applied an Actical instead of an ActiGraph applying different sedentary time and physical activity intensity cut-points thus hindering an effective comparison with our studies most of whom used an ActiGraph. While majority of the studies used an ActiGraph (13 out of 14), application of different models of ActiGraph may have resulted in different outcomes thus rendering an effective comparison between the studies difficult. Lastly, it is important to understand that South Asians are a diverse group originating from seven countries in the sub-continent region with strong within sub-group differences based on religion, language, culture and national origin. Studies originating from UK have highlighted significant differences among Pakistanis, Bangladeshis and Indians in physical activity levels and associated risk of coronary heart disease and cardio metabolic syndrome. Therefore, there needs to be some caution in generalizing results of these studies to all South Asians sub-groups.

## 5. Conclusion and future implications

Our review identifies a substantial gap that exists in South Asian population level studies based on objective measurement of sedentary time and physical activity via activity monitors and suggests some important avenues for future research. With South Asians being one of the fastest growing ethnic groups in both Canada and USA, [75,76], the CHMS as well as NHANES need to include nationally representative samples of this ethnic minority that has been identified at a high risk for chronic disease. In the meantime, we need methodologically strong studies based on randomly selected samples so that their findings can be generalized to the South Asian population at large. Furthermore, due to a considerable heterogeneity that exists within South Asians, future studies also need to investigate sub-group differences to ensure development of effective, culturally sensitive public health interventions targeting all sub-groups within this ethnic group.

## Supporting information

S1 Checklist PRISMA 2009 checklist. (DOC)

S1 File (DOCX)
