# Supplementary material for: Objective measurement of physical activity and sedentary behavior among South Asian adults: A systematic review
Source: PLoS One. 2020 Aug 5;15(8):e0236573. doi: 10.1371/journal.pone.0236573 (PMC7406035; doi:10.1371/journal.pone.0236573)
Supplement: S1 File — (DOCX) [file pone.0236573.s002.docx]

**Adapted NOS Scale (and coding manual) for quality assessment of studies using motion sensors to measure physical activity and sedentary time.**

**SELECTION**

1. Representativeness of the sample (Maximum number of stars 3)
2. Truly representative of the target population (random sampling) **
3. Somewhat representative of the target population (non-random sampling) *

Item is assessing how representative the sample is of the target population. For example, depending upon study focus, a random sample of South Asians recruited from South Asian community centers, ethnic events, SA market places, neighborhoods with a high density of SA’s or places of worship (Gurdwaras, temples, Mosques) would be more representative of the SA community as a whole rather than a sample of SA’s recruited from a specialty clinic or university.

1. Sample size: (Maximum number of stars 1)
2. Justified and Satisfactory *
3. Not justified

The study needs to justify its sample size calculation. If the sample size is too small, the study may fail to detect important effects or associations. Since for this review, we are primarily looking at baseline prevalence of PA, with a smaller sample size, study precision will be compromised.

Maximum number of stars that can be allotted is one.

3a) Response Rate for the study and Non-respondents: (Maximum number of stars 2)

1. Response rate is satisfactory *
2. Comparability between the responders and the non-responders has been established*

This refers to response rate for the study – how many potential respondents were approached and how many of those who were considered eligible, agreed to participate. A response rate of 70% or higher will be considered satisfactory. A study also needs to mention if there were any differences ( such as demographics etc.) between responders and non-responders.

3b) Compliance Rate for the accelerometer trial and Non-respondents: (Maximum number of stars 3)

1. Response rate is satisfactory * ( two stars if response rate is 100%)
2. Comparability between the responders and the non-responders has been established*

For the purpose of this review, this response rate refers to 3 to 7 day accelerometry trial component with the proportion of participants meeting the minimum number of valid days’ requirement. A response rate of 70% (meaning that at least 70% of the participants returned their accelerometer meeting the minimum valid day’s requirement) or above will be considered satisfactory.

To rule out non-response bias, it is important that the study provides information on non-responders - those who did not meet the minimum number of valid days’ requirement for the accelerometry trial – and whether there were any significant differences between those who responded and those who did not. For example, were the non-responders or those who did not meet the valid day’s requirement older/younger, of a higher BMI, low education attainment, low income level etc.?

**COMPARABILITY:** When Physical activity levels in South Asians are being compared with other ethnic groups such as White (Maximum number of stars 2)

1. The study controls for the most important factor *
2. The study controls for any additional factor *

This item will only apply to those studies where physical activity levels among South Asian ethnic group are being compared to another group/s (e.g. White). It is important that studies control for important factors like age (since SA immigrants tend to be relatively younger than Whites) as well as income and education – factors known to influence lifestyle behaviors.

Maximum number of stars that can be allotted to this item is two – one for adjusting for the most important factor and one for any additional factor.

**ACCELEROMETRY DATA PROCESSING/ANALYSIS DECISIONS (Maximum number of stars 9)**

The following 9 items under this sub-heading are important decisions that impact the quality of accelerometry studies. Inclusion of any of these items in the study is eligible for one star each.

1. Authors have asked the participants to wear the accelerometer for a minimum of three to four days *

Studies show that a minimum of 3 to 7 days of monitoring period with an accelerometer is sufficient to assess habitual physical activity levels. For this review, a study with at least a minimum of 3-4 days of monitoring will be considered meeting the minimum quality standard.

1. Study specifies whether accelerometer is to be worn during waking hours only or during sleep as well *

| 1. Placement of accelerometer is specified *   If the accelerometer is worn on the hip, arm, thigh etc.   1. Wear and non-wear time calculation is specified *   It is important that the researchers identify wear and non-wear time. Mostly studies define a valid day as 10 hours or more of wear time and wear time is defined by subtracting non-wear time from 24 hrs.  Non-wear time needs to be differentiated from period of inactivity – that is, when the monitor is being worn but no activity is being recorded because of the person being sedentary. Most studies on adults define non-wear as a period of at least 60 minutes of consecutive zeros with allowance of 1 to 2 minutes of interruptions or counts between 0 and <100.   1. Required hours of wear time per day to be considered a valid day are specified *   For example, most studies consider 10 or more hours of wear time a day as a valid day   1. Minimum number of valid days of data to be included in analysis is specified *   For example, a minimum of four days of valid data will be required before the file is included for analysis   1. Epoch length is specified *   Accelerometers function by integrating a filtered digitized acceleration signal over a  user-specified time interval, commonly referred to as an epoch. At the end of each  epoch, the summed value or activity count is written to memory. If the volume of  activity (activity counts over a specified time frame) is the outcome of interest, epoch  length is not an issue. However, if one applies cut points to determine the amount of  time spent in different levels of intensity, then the choice of epoch length may affect the  study results and should be carefully considered before collecting data in the field.  Outcome will be different with the application of a 15 min vs 60 sec epoch. Researchers  therefore, need to specify what epoch length was applied.   1. Cut points used to classify various intensities of physical activity are specified *   Cut-points are the thresholds of activity counts used to categorize activity as sedentary light, moderate, vigorous or very vigorous physical activity. The choice of cut-points impacts PA outcome as well as comparability across studies. It is thus important for the studies to identify which cut points were applied.  For adult studies, most frequently used cut-points are those provided by Freedson:  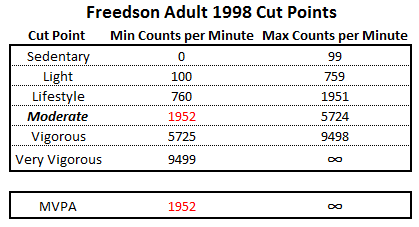   1. An activity diary/log is asked to be kept *   **OUTCOME:**   1. Assessment of the outcome (Maximum number of stars 3) 2. Objective (activity monitor) *   Outcome (in this case physical activity levels and sedentary time) is being measured objectively via a motion sensor.   1. Self-report and objective **   Outcome (in this case physical activity levels and sedentary time) is being measured objectively via a motion sensor as well as through self-report which makes it possible to corroborate results.   1. Statistical test (Maximum number of stars 3) 2. The statistical test used to analyze the data is clearly described and appropriate* 3. Outcome measures are presented with confidence intervals/Standard Deviations and p-value * 4. Important factors (confounders like age, income, education, wear time) have been controlled for * |
| --- |
|  |
|  |
|  |
|  |

.
